# Oil-based vitamin E oral spray for oral health in pregnancy

**DOI:** 10.2144/fsoa-2021-0095

**Published:** 2022-03-01

**Authors:** Sabrina Reppuccia, Felice Crocetto, Elisabetta Gragnano, Pietro D'Alessandro, Martin Vetrella, Gabriele Saccone, Bruno Arduino

**Affiliations:** 1 Department of Neuroscience, Reproductive Sciences & Dentistry, School of Medicine, University of Naples Federico II, Naples, Italy

**Keywords:** NICU, periodontal disease, pre-eclampsia, preterm birth, supplements, vitamin

## Abstract

**Aim:**

To assess the efficacy of vitamin E oral spray in pregnancy.

**Materials & methods:**

This was a retrospective study aimed to evaluate efficacy of vitamin E oral spray (vitamin E acetate in a medium chain tryglicerides vehicle – patented formulation) starting from the first trimester of pregnancy, with a control group.

**Results:**

A total of 100 women were included in the study and were compared with a matched control group. Only 25/200 women reported to have at least one teeth cleaning during pregnancy. Women who received the oral spray had a significantly lower risk of preterm birth compared with the control group, and lower risk of periodontal diseases.

**Conclusion:**

Use of oil-based vitamin E oral spray in pregnancy is associated with a decreased risk of periodontal diseases and therefore preterm birth.

## Plain language summary

Periodontitis, an inflammatory disease caused primarily by gram-negative bacteria that destroy tooth-supporting connective tissue and bone, is associated with an increased risk of preterm birth, as well as low birth weight and pre-eclampsia. Use of oil-based vitamin E oral spray in pregnancy is associated with a decreased risk of periodontal diseases and therefore preterm birth.

Preterm birth, defined as delivery before 37 weeks of gestation, is the leading cause of perinatal morbidity and mortality [1]. Different strategies have been adopted for prevention of preterm birth, including progesterone, cerclage, cervical pessary, as well lifestyle modifications [2–24].

The etiology of preterm birth and preterm labor is not completely understood [25]. According to Romero *et al.* preterm labor should be seen as one syndrome with many causes associated with intra-amniotic infection, decidual senescence and breakdown of maternal–fetal tolerance [25]. More than 30% of all preterm labors is linked to intra-amniotic infection [25]. Micro-organisms may gain access to the amniotic cavity via many pathways, including vagina and cervix, peritoneal cavity, invasive procedures, as well as by hematogenous dissemination through the placenta [26]. Therefore, any focus of infection may lead, hypothetically, to preterm labor and preterm birth.

Recently, several studies have reported a significant association between oral hygiene and preterm birth by using image analysis tools to quantify dental plaque biofilm [27]. Periodontitis, an inflammatory disease caused primarily by gram-negative bacteria that destroy tooth-supporting connective tissue and bone, is associated with an increased risk of preterm birth, as well as low birth weight and pre-eclampsia [28]. Research suggests that the bacteria that cause inflammation in the gums can actually get into the bloodstream and target the fetus, potentially leading to premature labor [27,28].

## Objective

The aim of the study was to assess the efficacy of oil-based vitamin E oral spray for oral health in pregnancy in reducing the risk of preterm delivery.

## Materials & methods

### Study design

This was a retrospective cohort study using data of women at risk of preterm birth or prior preterm birth, or history of prior cervical surgery. Women with multiple gestations were excluded. In this study, we retrospectively analyzed data on oral health and use of fluoride toothpaste, mouthwash and flossing. We planned to compare women who used oil-based vitamin E oral spray (vitamin E acetate in a medium chain tryglicerides vehicle – patented formulation) with a control group who did not.

### Inclusion & exclusion criteria

Inclusion criteria were singleton gestations and at least one risk factor for preterm birth including prior preterm birth, short mid-trimester transvaginal ultrasound cervical length, obesity or prior cervical surgery.

Exclusion criteria were multiple gestations, and women with fetal abnormalities. Women with placenta previa/accreta were also excluded [29–31].

### Intervention & control group

Women in the intervention group used vitamin E acetate (in a caprylic/capric triglycerides vehicle) spray. Women used the therapy at least daily during the pregnancy. Women in the control group did not receive the oil-based vitamin E oral spray.

### Primary & secondary outcomes

The primary outcome was preterm birth. The secondary outcomes were pre-eclampsia, perinatal death, admission to neonatal intensive care unit and birth weight.

### Study definition

Preterm birth was defined as delivery before 37 weeks. Pre-eclampsia was defined as a blood pressure elevation (≥140/90 on two occasions 4 h apart or ≥160/110 once), after 20 weeks of gestation, with proteinuria (≥300 mg on 24 h protein or >0.3 protein/creatinine ratio) or any of the following if proteinuria not present: platelets <100,000; creatinine >1.1 (or doubling of creatinine in absence of other renal disease); doubling of aspartate aminotransferase (AST) or alanine aminotransferase (ALT). Perinatal death was defined as either stillbirth (i.e., fetal death after 20 weeks of gestation) or neonatal death (i.e., death of a live-born infant within the first 28 days of life).

### Statistical analysis

Statistical analysis was performed using Statistical Package for Social Sciences (SPSS) v.19.0 (IBM Inc., NY, USA). Data were shown as means ± standard deviation or as a number (percentage). Categorical variables were compared using the chi-square test with continuity correction. Within-group comparison was undertaken using t*-*test to test group means by assuming equal within-group variances. Normality assumption check of continuous variables was performed using the Kolmogorov–Smirnov test.

A p-value < 0.05 was considered statistically significant.

## Results

A total of 100 women who received the oral spray were included in the study and were compared with a matched control group (n = 100) who did not. The two groups were similar in terms of maternal demographics (the available maternal demographics are reported in Table 1). Overall, only 25/200 women reported to have at least one professional teeth cleaning during pregnancy. Use of fluoride toothpaste, mouthwash and flossing was fair, with an overall rate of 35, 22 and 13%, respectively. The vast majority of women in the intervention group used the oil-based vitamin E oral spray twice per day (67/100), while 33/100 used it about once per day. About 65% of the women started the therapy in the first trimester, 30% in the second trimester and all through the gestation and 5% in the third trimester of pregnancy. Out of the 65 women who started the therapy in the first trimester, 49 used the oil-based vitamin E oral spray all through the gestation, nine only in the first and second trimester and seven only in the third trimester.

**Table 1.** Available maternal characteristics of the included women.

	Oral spray (n = 100)	Control (n = 100)
Age (years)	28.5 ± 6.1	29.9 ± 6.4
Prior preterm birth	59 (59%)	51 (51%)
Prior cervical surgery	7 (7%)	11 (11%)
TVU CL ≤25 mm	44 (44%)	53 (53%)
BMI >30	17 (17%)	21 (21%)

Data are shown as number (percentage) or as mean ± standard deviation.

TVU CL: Transvaginal ultrasound cervical length.

Table 2 shows the primary and secondary outcomes. Women who used the oral spray had a significantly lower risk of preterm birth compared with the control group, and higher birth weight of about 160 g. Rate of periodontal diseases was 55% in the control group and 33% in the intervention group.

**Table 2.** Primary and secondary outcomes.

	Oral spray (n = 100)	Control (n = 100)	p-value
Preterm birth at less than 37 weeks	15 (15%)	27 (27%)	**0.04**
Pre-eclampsia	5 (5%)	6 (6%)	NS
Perinatal death	0	0	-
Birth weight, g (mean ± SD)	3,105 ± 748	2,945 ± 860	Mean difference 160 g (-63.39 to 383.39)
Admission to NICU	6 (6%)	8 (8%)	NS

Data are shown as number (percentage) or as mean ± standard deviation.

Boldface data is statistically significant.

NICU: Neonatal intensive care unit; NS: Non significant; SD: Standard deviation.

## Discussion

This study aimed to assess the efficacy of oil-based vitamin E oral spray for oral health in pregnancy. We found in women at risk of preterm birth that use of oil-based vitamin E oral spray during pregnancy was associated with a decreased risk of preterm birth. The decreased preterm birth rate may be associated with the decreased risk of periodontal diseases in the intervention group.

The main limitations of the study were the small sample size, and the nonrandomized approach. Given the retrospective study design, we cannot evaluate the compliance with the therapy. Moreover, we did not evaluate the presence of gingivitis or periodontal disease in the participants. The information about oral health was self-reported by the women leading to possible reporting bias and recall bias, potentially affecting our study findings. The major limitation of this study was the absence of randomization. Another limitation was the asimmetric gestational ages at assumption of the intervention.

### Implication

Preterm birth is defined as delivery before 37 week of gestations [32]. Common causes of preterm birth include multiple pregnancies, infections and chronic conditions such as diabetes or hypertension. Recently, many studies have focused on the role of periodontitis as risk factor for preterm birth [33]. Periodontal diseases increase levels of circulating IL-1β, IL-6, IL-8, IL-17 and TNF-α [34], interleukins associated with systemic inflammation and preterm birth [35].

Although intervention trials reported contradictory results [36,37], oral health is an important part of prenatal care and should be supported before, during, and after pregnancy [38]. Oral health self-care practices based on procedures, such as flossing or dental water jet may reduce the risk of periodontal disease [39]. Available evidence suggests that self-administered oral sprays are a safe and effective method for oral health [40], but evidence are limited on effectiveness for control of plaque and gingival inflammation [41], as well as in improving pregnancy outcome in pregnant women [42].

Vitamin E is a potent antioxidant with anti-inflammatory properties [43,44]. Vitamin E supplementation has been shown to enhance the function of the immune system and reduce risk of infection, particularly in susceptible individuals, such as older individuals and pregnant women [44]. A recent study also showed that vitamin E level in pregnant women was associated with composite adverse perinatal outcomes [45].

In our study we investigated the role of oil-based vitamin E oral spray for oral health in pregnancy. We found that in a small cohort of pregnant women, with risk factors for preterm birth and with a low compliance for professional teeth cleaning, use of vitamin E oral spray is associated with a reduction in the rate of preterm delivery.

## Conclusion

In summary, daily use of oil-based vitamin E oral spray in pregnancy may be associated with a decreased risk of preterm birth.

**Summary points**

- Periodontitis is associated with an increased risk of preterm birth.
- Use of oil-based vitamin E oral spray in pregnancy is associated with a decreased risk of periodontal diseases and, therefore, preterm birth.
- We suggest to offer to women at risk of preterm birth or periodontal diseases the use of daily oil-based vitamin E oral spray.
